# Medical Digital Twin: A Review on Technical Principles and Clinical Applications

**DOI:** 10.3390/jcm14020324

**Published:** 2025-01-07

**Authors:** Mario Tortora, Francesco Pacchiano, Suely Fazio Ferraciolli, Sabrina Criscuolo, Cristina Gagliardo, Katya Jaber, Manuel Angelicchio, Francesco Briganti, Ferdinando Caranci, Fabio Tortora, Alberto Negro

**Affiliations:** 1 Department of Advanced Biomedical Sciences, University “Federico II”, Via Pansini, 5, 80131 Naples, Italy; francesco.briganti@unina.it (F.B.); fabio.tortora@unina.it (F.T.); 2 Department of Precision Medicine, University of Campania “L. Vanvitelli”, 80131 Caserta, Italy; fpacchiano1@gmail.com (F.P.); ferdinando.caranci@unimol.it (F.C.); 3 Department of Radiology, Massachusetts General Hospital, Boston, MA 02115, USA; sfazioferraciolli@mgh.harvard.edu; 4 Pediatric Imaging Research Center and Cardiac Imaging Research Center, Massachusetts General Hospital, Harvard Medical School, Boston, MA 02115, USA; 5 Pediatric University Department, Bambino Gesù Children Hospital, 00165 Rome, Italy; sabrinacriscuolo93@gmail.com; 6 Pediatric Department, Ospedale San Giuseppe Moscati, 83100 Aversa, Italy; cristy.g@hotmail.it; 7 Department of Elektrotechnik und Informatik, Hochschule Bremen, 28199 Bremen, Germany; katyajaber@hotmail.de; 8 Biotechnology Department, University of Naples “Federico II”, 80138 Napoli, Italy; manuel.angelicchio@gmail.com; 9 Neuroradiology Unit, Ospedale del Mare ASL NA1 Centro, 80145 Naples, Italy; alberto.negro@hotmail.it

**Keywords:** digital twin, healthcare, oncology, cardiology, neuroscience

## Abstract

The usage of digital twins (DTs) is growing across a wide range of businesses. The health sector is one area where DT use has recently increased. Ultimately, the concept of digital health twins holds the potential to enhance human existence by transforming disease prevention, health preservation, diagnosis, treatment, and management. Big data’s explosive expansion, combined with ongoing developments in data science (DS) and artificial intelligence (AI), might greatly speed up research and development by supplying crucial data, a strong cyber technical infrastructure, and scientific know-how. The field of healthcare applications is still in its infancy, despite the fact that there are several DT programs in the military and industry. This review’s aim is to present this cutting-edge technology, which focuses on neurology, as one of the most exciting new developments in the medical industry. Through innovative research and development in DT technology, we anticipate the formation of a global cooperative effort among stakeholders to improve health care and the standard of living for millions of people globally.

## 1. Introduction

Digital twins (DTs) are increasingly being used in many different industries; in this regard, the health industry has seen a recent increase in DT use. In the end, the idea of digital health twins promises to improve human life by revolutionizing illness prevention, health maintenance, diagnosis, management, and treatment. By providing crucial data, a robust cyber technology infrastructure, and scientific expertise, big data’s exponential growth, in conjunction with continuous advancements in data science (DS) and artificial intelligence (AI), has the potential to significantly accelerate DT research and development. Despite the military and industry’s numerous DT programs, applications in healthcare are still in their early stages. This article’s aim is to introduce this novel technology, which is among the most promising to have been released recently in the medical profession, with an emphasis on neuroscience. This narrative review includes articles obtained from the main search engines for scientific papers (Pubmed, Google Scholar, Scopus and IEEE Xplore) with key words such “Digital Twin”, “Digital Twin and Medicine”, “Digital twin and neuroscience” and “Digital twin limitations”. Through groundbreaking research and development in DT technology, we expect stakeholders to work together internationally to enhance health care and the standard of living for millions of people worldwide.

## 2. Methodology

We conducted a thorough literature study on this subject by looking through earlier published articles. We conducted literature searches on Scopus (https://www.scopus.com), PubMed (https://pubmed.ncbi.nlm.nih.gov), and Google Scholar (https://scholar.google.com) using the following keywords: “Digital Twin” AND “Medical” AND/OR “Technical Principles” AND/OR “Clinical Applications” AND/OR “Healthcare” AND/OR “Oncology” AND/OR “Cardiology” AND/OR “Neuroscience”.

## 3. Origin and Conceptualization of the Digital Twin

The “twin strategy” concept was first employed by NASA around the 1960s and was used during the Apollo 13 mission to bring astronauts back to Earth following a spacecraft malfunction [1]. In summary, this technology involved rebuilding a spacecraft identical to the one launched into space. In this way, it was possible to reproduce and then handle possible malfunctions by first practicing on the ground model, which was built to mirror the in-orbit model [2]. However, the first mention of the term “digital twin” was employed by Grieves in 2003 in an industrial context about product lifecycle management [2,3,4,5]. Later, in 2012, DT became the main technology for future vehicles, according to a paper issued by NASA and the U.S. Air Force in the person of Vickers [1,2]. Since then, DT has been used in various industries, including automotive, oil, and gas, and research on it in aerospace has grown. Real-time monitoring systems to identify oil and gas pipeline breaches are among examples of the use of DT, as are traffic and logistics management [6]; however, it has only been in recent years that DT has been introduced in the healthcare and medical fields, as preliminarily reported in the journal Nature in 2024 [7].

The growth of DT will be accelerated by the accessibility of low-cost technologies and the extraordinary success of technologies like artificial intelligence and machine learning (ML) [5]. Grieves [4] suggests framing DT in three dimensions: as a physical thing, a digital replica, and a link connecting the two.

Thus, it is recognized as a technology that enables a virtual representation that engages with the physical thing throughout its life cycle and offers data for managing, predicting, and assessing its attributes. In order to forecast, assess, and manage the physical subject’s health, DT would enable the representation of a virtual person in the healthcare industry [2,8,9,10,11,12,13,14].

Some research [15] has claimed that, when properly integrated with data, DT can provide real-time information for better decision making and can forecast the future behavior or development of an object or subject, in addition to providing a static image of that object or topic. In a perfect world, a DT would exhibit behavior and an appearance that is identical to that of a physical thing, with the extra advantage of being able to predict the future. Additionally, this provides the option for users to engage with the object in person through an avatar [6,7,16,17,18,19,20,21,22,23,24,25,26,27,28]. In this regard, in fact, it is possible to find in the literature different types of DT, which constitutes a clear evolution of the concept in recent years.

Initially, the so-called static twin was proposed, which represents a digital replica of a physical system. New variations have since surfaced, such as the “shadow twin”, “mirror twin”, and, more recently, the “intelligent twin” [29,30,31].

- Based on historical or infrequently updated data, a static twin model may depict the starting condition of a real thing and has solely static properties. While a functional twin, also known as a mirror twin, continuously receives real-time data from the physical asset, guaranteeing that the digital model evolves in parallel, this digital model does not evolve in real-time [32]. There are few instances of mirror twins being used for surgical planning in the medical industry.
- The self-adaptive twin, also known as the shadow twin, is a functional twin that can gather data in real time and update the model by monitoring changes and interacting with an actual system, organism, or item [33]. Several instances of shadow twins have been used in the medical industry for medication and biomarker development.
- Thanks to machine learning and artificial intelligence programs, the most sophisticated kind of DT is the intelligent, self-adaptive twin, often referred to as extended DTs, cognitive DTs, or physical avatars, which have the capacity to learn, reason, know, act, and communicate with other twins. Examples of individualized medicine in healthcare have been utilized [31,34].

The emerging field of precision medicine represents fertile ground for the application of DT in areas such as cancer care. In order to undertake individualized diagnosis, treatment planning, care, and survival, it can be utilized to replicate an individual’s health condition. Every individual is different; thus, while creating patient-specific DTs, it is critical to gather detailed digital phenotypes. The usefulness of the digital twin across a wide range of healthcare industry sectors is acknowledged in this review [31,34].

## 4. Digital Twin in Healthcare

**Figure 1:**
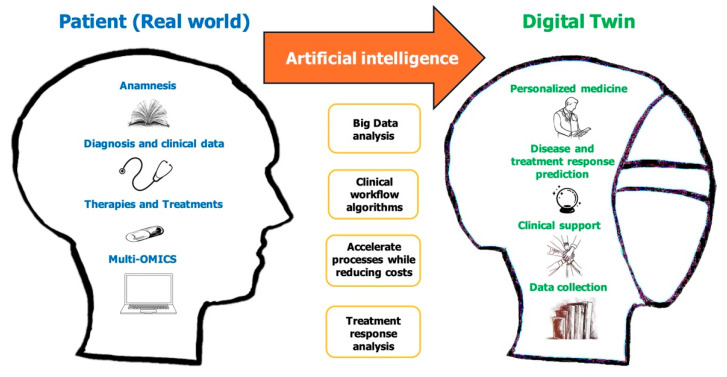
Digital twin for health (DT4H) envisioned.

A DT for healthcare, according to Katsoulakis [31] is a virtual representation of an individual that enables early intervention and prevention, the monitoring and prediction of a health trajectory, and the dynamic modeling of a possible treatment approach. Multiscale modeling of multimodal data, such as genetic, molecular, environmental, social, radiological, and clinical aspects, might be used to achieve this. (Figure 1).

Thus, DT technology in the healthcare sector consists of a physical object, a virtual replica, and a link between the two to enable a real-time bidirectional impact on each other. The constantly changing relationships between the physical being and its digital duplicate may occur at microscopic or macroscopic lengths, or throughout an individual’s life, from conception to death. The five characteristics (5I) of a successful DT are that they be impactful, informative, interactive, personalized, and linked. To help with better treatment outcomes and fewer side effects, a patient-specific DT may be created via DT simulation, prediction, and analysis. On the other hand, DT modeling may be enhanced, validated, and benchmarked using real patient data [30,31,32,33,34,35,36].

Personalized medicine has made extensive use of AI/ML algorithms recently. A system (a person, an organ, an illness, or a medication) has to be unique, linked, interactive, impactful, and personalized in order to be classified as a digital twin (DT). It also needs to have the three essential elements—a physical entity, a virtual duplicate, and a link between the two. The subtleties and differences between different virtual models are crucial, particularly when taking into account elements like applications, data sources, interactions, and visualization techniques [31].

For instance, comprehensive imaging data are mostly used in the development of DTs that simulate particular organs, like the living heart model. In sharp contrast, illness models used to promote precision medicine are based on a comprehensive combination of clinical and molecular profiling data. These models’ range of applications matches the diversity of their data sources. DTs that are particular to an organ, such as those that mimic cardiac activity, are essential for forecasting how mechanical medical devices, like pacemakers and stents, will behave. Conversely, illness models offer important information on how well pharmacological treatments work, especially in terms of how medications interact with intricate biological systems. Apart from these distinctions, there are notable variations in the computational analysis utilized in every category of model. This variation shows the specialized nature of the analysis required as well as the distinct demands and objectives of each model. To fully utilize DT potential to enhance patient outcomes and progress medical science, it is imperative to comprehend these distinctions [31].

Considering all of this, it is nevertheless predictable that, when considering the potential of DT, the fields of medicine and healthcare stand to gain the most from the idea [5]. We anticipate this due to a number of factors, such as the steadily growing number of smart portable devices, the systematic archiving of big data about individuals and cohorts, and the enormous and ongoing expansion of healthcare knowledge (e.g., differentiated diagnoses, more personalized therapies, interaction risks, active ingredients).

The development of these technologies is being driven by the need for assistance for medical professionals who are constrained by everyday events that render people less effective than computers, particularly during times of intense emotion and stress.

Personalized and targeted treatments are also becoming ever more necessary [37]. Therefore, the primary force behind smart and networked health is the fusion of technology and medicine.

In this context, the statistical modeling of large amounts of data is a special challenge. Classical techniques that examine relationships between individual variables and a diagnosis or the evolution of an illness are limited by the vast number of statistical tests required, in addition to their inability to uncover complex interactions between numerous components and modalities in real time.

Furthermore, even the smallest impacts surpass the significance barrier if the entire population—rather than just samples, as is typically the case—is included in our statistical examination. As a result, the relationship between significance and (clinical) relevance disappears [2].

Therefore, ML is essential to generating a clear clinical benefit.

Algorithms that can learn to accomplish a particular job on their own using data are used in ML. These algorithms can produce novel solutions to challenging tasks and challenges as they do not require explicit programming. ML techniques are more effective for spotting patterns, generating features, and making predictions from large, complex, and heterogeneous data because they can be applied to a variety of data types, enable analysis and interpretation across complex variables, and are generally more generalizable, even though traditional statistical methods can also be used to find correlations and generate predictions. Thus, ML techniques may be applied in highly novel fields, including drug discovery, omics, radiology, and individualized therapy, and they can also be utilized to supplement and expand established statistical approaches [38,39].

ML is a crucial part of a modern DT, allowing one to take advantage of the synergies that result from their combination [40] and also because a DT in healthcare is a “virtual mirror of ourselves that allows us to simulate our personal health history and health status using data-driven analytical algorithms and theory-based physical knowledge” [41]. Stated differently, a DT combines the deductive approach—mechanistic models that integrate multiscale knowledge and data—and the inductive approach—statistical models that learn from data—to produce accurate forecasts of the paths to maintain or recover health [42].

Without putting patients in danger, the capacity to simulate and model pharmaceutical therapies and medical equipment on computers enables more rapid and convenient development than in real-world settings [30,42]. This is known as “making mistakes on computer models instead of people” [43].

**Figure 2:**
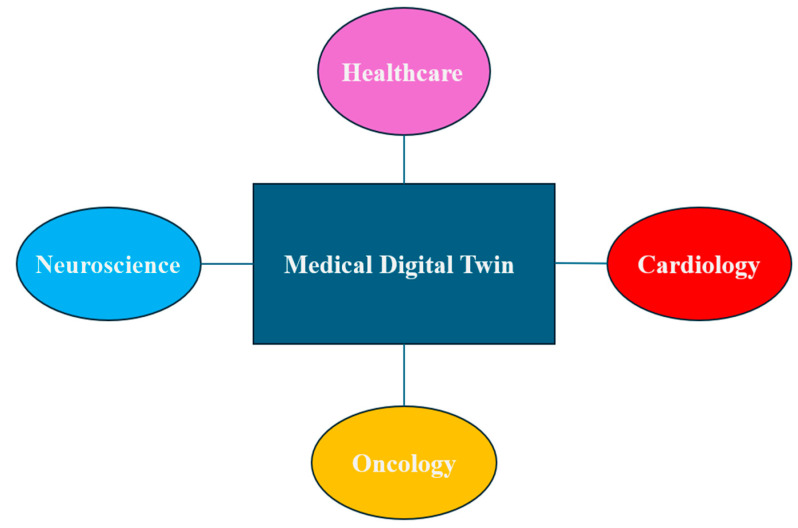
The outline of our paper. In particular, we describe the main application field of the medical digital twin with particular regard to healthcare, cardiology, oncology and neuroscience.

We are still in the early stages of DT usage in medicine. Only a few medical specialties [44,45,46,47,48,49,50,51,52,53,54,55,56,57,58,59], especially oncology and cardiology, have used DTs to date. The aim of our paper is to present an overview of DT clinical applications with a special focus on neuroscience. The literature has not yet examined this subject in an organic manner (Table 1 and Figure 2).

## 5. Clinical Application in Oncology

Among the most studied uses of DT are those in oncology that are aimed at a precise, highly personalized and dynamic approach. The creation of digital replicas allows a comprehensive understanding of individual cancer cases, enabling simulation, analysis and prediction of cancer progression and treatment outcomes in a virtual environment. From the virtual replica, data are extended to the real world [60,61,62,63]. To do this, careful selection of big data and meticulous integration of diverse patient data, such as clinical, radiological, and genetic information, appears crucial. Thus, optimization of omics technologies and integrations between radiomics and genomics comes to the aid of such technology. This rich data set forms the basis of the DT, enabling simulation of tumor behavior and evaluation of potential treatment strategies.

The various dynamical systems models are critical for modeling the molecular interactions within tumor cells that ultimately determine cellular phenotypes. In addition, ML algorithms help to identify patterns and correlations in large datasets, helping to predict tumor grade/behavior and response to treatments with greater accuracy and efficiency [64,65,66].

DT, in addition to providing the current phenotype status of an individual with cancer, can also dynamically represent the object/subject allowing accurate monitoring of tumor progression and evaluation of treatment responses. This continuous assessment allows for real-time, data-driven changes to the treatment plan, ensuring the most effective and personalized care delivery.

There are several DT projects active right now. Using a partial differential equation model of breast tissue calibrated using patient-specific data from magnetic resonance imaging (MRI) and quantitative positron emission tomography, Wu and colleagues have established a project [67] that tries to forecast the course of breast cancer. Model parameters that reflect the characteristics of cell migration and proliferation of tumor cells specific to the given tumor are derived from the images.

The effectiveness of immunotherapy or the impact of medication therapies may both be predicted using this model [68]. Within the expanding field of mathematical neuro-oncology, a similar strategy has shown promise in treating patients with glioblastoma, a kind of extremely aggressive brain tumor with a dismal prognosis [69]. MRI is also used in this DT [70]. For example, the Swanson lab used two parameters in the partial differential equation, one representing the rate of tumor proliferation and the other the rate of cell migration, which may be estimated from the patients’ MRI sequences. These two criteria are unrelated to one another, as tumor cells have been shown to either divide or migrate through the tissue, but not both at the same time. These patient-specific data indicate the possibility of anticipating the true tumor extent in order to assess the best surgical resection for the best survival.

Tumor cells penetrate surrounding brain matter in a diffuse way that is not well recorded by imaging, which in turn makes it impossible to precisely establish the exact size of the region of the brain harboring the tumor cells using MRI images alone. Additionally, altered versions of the DT can forecast the results of certain therapies [71]. Despite significant advancements in this field, several significant obstacles remain to be overcome. These include insufficient patient data, inadequate mechanistic comprehension of the various tumor subtypes, and the effectiveness of an ever-growing supply of anticancer medications, which hinders model-based prediction of the correct interventions at a given disease stage.

## 6. Clinical Application in Cardiology

Cardiology is another newer application area for DT. There is a great deal of promise in developing virtual heart models to enhance cardiovascular disease diagnosis, treatment, and prognosis. A cardiac DT usually consists of three basic parts: modeling and simulation (based on anatomy and physiology); data collecting (imaging, electronic health record, genetic data); and clinical decision making. DTs are essential in the study of arrhythmias in cardiology. Specifically, the use of DTs to guide the therapy of arrhythmias by catheter ablation and the prediction of sudden cardiac death owing to arrhythmias in a variety of conditions. The prevalence of arrhythmia-related sudden cardiac death is rising worldwide, and a significant unmet therapeutic need is the precise, individualized evaluation of mortality risk. Cardiologists have made significant progress in anticipating patients with ischemia (induced by coronary atherosclerosis) and non-ischemic cardiomyopathies regarding their risk of sudden death [7].

In order to assess the risk of infarction-related ventricular arrhythmias and sudden death in a cohort of patients following ischemia, Arevalo and colleagues’ study [72] showed the first application of DT derived from contrast-enhanced MRI. Using the data from heart MRIs, they built a 3D computer model of 41 patients’ hearts after myocardial infarction. Here, the scar was identified as well as the infarct border zone, while the model included the electrophysiological properties depending on the tissue, where myocites in healthy tissue were assigned human action potential dynamics, the border zone with the scar had the potential for extended duration and remodeled conductivities, and the scar was indicated as a mute area. Stimulating the digital heart in different areas was the next step to assess whether that patient could have a life-threatening arrhythmia. DT prediction performed better than any clinical risk assessment metric currently available, suggesting that DT can be used to predict whether prophylactic defibrillator implantation is necessary to avoid sudden death.

Penetrating adipose tissue is an additional component of a more sophisticated method for utilizing DT to evaluate the arrhythmia likelihood of patients with past ischemia [70]. The therapeutic value of cardiac DT investigations in pediatric patients with acute myocarditis [71] and corrected tetralogy of Fallot [73], for the risk prediction of sudden cardiac mortality in patients with non-ischemic cardiomyopathies, has been established. Additionally, DT technology has significantly outperformed existing clinical risk predictors in the prediction of arrhythmia in hypertrophic cardiomyopathy [74], a prevalent hereditary illness characterized by thickening of the heart muscle. Another non-ischemic cardiomyopathy is cardiac sarcoidosis, an inflammatory condition linked to a high risk of sudden mortality and challenging risk assessment.

DT and ML are used in a two-step prediction method that Shade at al have developed [75]. A collection of clinical biomarkers and the multi-disciplinary team simulation results were loaded into a supervised classifier.

Lastly, a recently developed genotype-specific cardiac DT (Geno-DT) method has been used to predict arrhythmic circuits in individuals with various genotypes of arrhythmogenic right ventricular cardiomyopathy (ARVC) [76]. This method has been seen to show that different ARVC genotypes have different underlying arrhythmic processes. More therapeutic accuracy in the clinical situation has been shown to be possible with the Geno-DT technique, which might result in more individualized therapy plans for ARVC. Currently, managing arrhythmias involves the use of catheter ablation. In order to stop the arrhythmia creator, radiofrequency radiation is delivered to particular places using catheters that are inserted into the heart chambers. However, pinpointing these precise regions inside the heart is challenging, and ablation targets are frequently incorrect, leading to the emergence of additional arrhythmias following ablation.

By enabling noninvasive localization of ablation targets, personalized DT technology has significantly increased ablation accuracy [77,78,79,80,81]. Several years later, DTs have predicted not only the ablation targets for the first treatment, but also the ablation targets for follow-up surgeries. The potential to design various atrial fibrillation care methods and even anticipate a patient’s risk of recurrence is a fascinating feature of tailored DTs. About 1–2% of people have atrial fibrillation, which is the most common arrhythmia in humans and affects the upper chamber of the heart. While not as harmful as ventricular arrhythmias, it is linked to a significant risk of stroke and a large expense of healthcare because of hospitalization of the patient.

The effectiveness of various ablation techniques has been examined in a number of atrial DT studies including individuals who exhibit a persistent type of the arrhythmia. Atrial DTs that represent the distribution of patient-specific atrial fibrosis have been developed as a result of the identification of atrial fibrosis as a substrate for atrial arrhythmias [82,83,84,85,86].

Yang et al. used a multi-layered DT model in their study to assess the impact of neuromodulation on cardiac activity, especially in relation to arrhythmias and possible treatment options. The autonomic nervous system’s (ANS) parasympathetic and sympathetic branches are both replicated in the model. The model includes conductance-based integrate-and-fire neurons, synaptic dynamics, and random intralayer connections in each layer.

The association and dissociation rates of noradrenaline with the β-adrenergic receptor were determined using an atomic simulation in order to capture molecular-level interactions. The Behar–Yaniv model of the rabbit sinoatrial node (SAN) was used to link pacemaker cells and contractile cells to the ANS model in order to evaluate variations in heart rhythm brought on by autonomic activity.

Insights into how autonomic imbalance raises the likelihood of arrhythmias and how neuromodulation might be targeted for their prevention and therapy were provided by the model’s performance, which closely matched experimental data [87].

Boyle et al. [88] started a prospective ablation study for patients who had individualized atrial DTs but were still experiencing persistent atrial fibrillation and fibrosis. The suggested DT ablation targets were utilized in that study to direct patient care. Eventually, atrial DTs found use in predicting the recurrence of atrial fibrillation, frequently in conjunction with ML or other technologies [89].

The above-discussed first accomplishments with cardiac DTs built from pictures and other health data have created new opportunities for the development and use of DTs in cardiology. The capacity to combine continuous data from many streams will be crucial in ensuring that the patient’s cardiac condition is continually reflected in the patient’s DT.

## 7. Clinical Application in Neurosciences

### 7.1. Multiple Sclerosis as Model of Chronic Disease Management

DT can significantly improve the management of chronic diseases such as multiple sclerosis (MS) by optimizing diagnosis, treatment, and management strategies. DTs are a ground-breaking tool for detailed clinical phenotyping, improved disease course prediction, and characterization [90]. The display of DTs at various phases of MS is supported by big data analysis utilizing machine learning (ML), which also makes additional therapeutic decisions possible. There are starting points and viewpoints even though there is currently a lack of evolved DTs. To determine the correlations between variables and disease development in MS clinical trials, Walsh et al. [91] employed an unsupervised machine learning model, a form of machine learning in which the algorithm learns patterns and structures from data that are not labeled or categorized. With the use of a conditional restricted Boltzmann machine (CRBM), the authors were able to generate digital subjects via the data of MS patients for whom the trajectories were statistically indistinguishable from patients from the placebo arm of an MS trial. This would be a useful element in the treatment of these patients, as it could help identify those who may have a remitting disease or not. Some authors, such as Wang et al., have developed virtual brain models based on real data from individuals, including MRI scans (which provide anatomical, structural, and functional information), EEG or MEG recordings, and clinical and genetic data from patients. Based on the collected data, they constructed a brain network connectivity model that represents both the strength and direction of connections between different brain regions. The authors applied mathematical models to describe how these regions communicate and exchange signals under baseline conditions, as well as in pathological states such as epilepsy, Alzheimer’s disease, multiple sclerosis, aging, Parkinson’s disease, and schizophrenia [92]. The development of mathematical models that can efficiently describe the functioning of brain networks presents significant challenges in terms of computational power. Marasco et al. introduced a linear mathematical framework known as the Adaptive Generalized Leaky Integrate-and-Fire (A-GLIF) model, capable of accurately capturing the nonlinear firing behavior of hippocampal CA1 pyramidal neurons and interneurons. Their model makes a notable contribution by achieving a high degree of accuracy in replicating the firing patterns of these networks while simultaneously reducing computational cost [93].

The future need for segmentation, the generation of volumetric meshes, and the mapping of data from 3D reconstructions often involves a multi-step process and the use of different tools. In the paper by Sainz-DeMena et al., the authors focused on the development of a Python library capable of generating 3D surface and volume meshes from 2D MRI images of patients with neuroblastoma [94].

Some authors, such as Dang et al., after developing a consensus on the treatment of patients with acute ischemic stroke through a DELPHI model that included specialists from various fields involved in the care of this patient population, will incorporate this consensus as the foundation for developing a digital twin model. This model will assist in responding to the clinical variables of these patients, while also incorporating patient-specific data [95].

The identification of epileptogenic networks is a critical component in the surgical planning of patients with drug-resistant epilepsy. Lemaréchal et al. aimed to evaluate the influence of varying spatial resolutions in computational models on the accurate identification of these networks. Specifically, they utilized a digital twin (DT) approach, known as the virtual epileptic patient (VEP), to estimate the brain regions responsible for seizure onset, referred to as the epileptogenic zone (EZN).

The authors compared two modeling techniques with different spatial resolutions: the high-resolution neural field model (NFM) and the lower-resolution neural mass model (NMM). Simulations of brain activity were conducted using the NFM, which consisted of 81,942 nodes, providing detailed spatial resolution. The simulated signals were then projected to stereotactic electroencephalography (SEEG) contact points. Subsequently, an inversion procedure was applied using the NMM, comprising only 162 nodes, to estimate the excitability of specific brain regions.

The results highlight the limitations of the NMM, including the oversimplification of local brain interactions, which led to an overestimation of brain excitability. Additionally, the NMM demonstrated reduced performance in identifying epileptogenic networks, particularly in more complex cases. These findings underscore the importance of employing higher-resolution models like the NFM to achieve greater accuracy in identifying epileptogenic zones, thereby improving clinical outcomes in presurgical evaluations for epilepsy patients [96].

A web-based DT platform with a transactional and analytical application was created [97] for MS diagnosis and rehabilitation. Nevertheless, the analytical application is still in its infancy and will require more study. Because MS is long-term and complex, a lot of multidimensional data need to be gathered and arranged in order to build DT-MS [88,89]. In-depth clinical phenotyping and DT data content in MS depend on patient physiological state data, which include structured clinical and paraclinical data [33,98]. Contextual elements, including lifestyle determinants, comorbidities [99], psychological aspects, emotions, and sociodemographic factors, should also be documented [100,101,102]. Structured clinical data include the gathering of patient history.

Tools like MSProDiscuss [103] and the Expanded Disease Disability Scale [104,105,106] and are used in attempts to standardize and measure neurological history in MS. To measure the multifaceted characteristics of MS, including walking function, fatigue, and cognition, additional clinical instruments have been developed [107,108]. A very sensitive functional evaluation of important MS functions is the Multiple Sclerosis Functional Composite (MSFC). Clinical phenotyping of MS in terms of disease activity or certain symptom phenotypes may be possible with these intricate data [109]. Nevertheless, DTs rely on data-driven methods and it is strongly advised to use a variety of clinical outcome metrics [102]. There are currently efforts to standardize the collecting of clinical data [110,111].

Para-clinical data, which range from routine laboratory measures to sophisticated immunological or neurological measures, are essential for the diagnosis, phenotyping, and monitoring of multiple sclerosis (MS) [112,113,114,115,116,117]. As the MS disease process takes place in the central nervous system (CNS), cerebrospinal fluid examination is very crucial [118,119]. Because it provides both qualitative and quantitative methods for MS diagnosis, CNS imaging has become more significant [120,121]. While quantitative data identify pathological alterations in tissues, qualitative data also help diagnose conditions more accurately by identifying certain biomarkers [122]. These findings offer a viable strategy for improving MS care via in in vivo observation [114]. Three-dimensionally resolved sequences serve as the foundation for computer-assisted image data analysis and volumetric measures, and standardization of MRI acquisition is crucial [123,124,125,126].

These developments might be easier to apply in clinical practice if they are incorporated into DT [127]. Because quantitative MRI produces many numerical results, it will make it possible to characterize brain tissue in depth. The massive amount of MRI data may be handled by artificial intelligence, a crucial component of DTMS [128]. MRI categorization is a crucial part of DTMS because it can characterize various MRI phenotypes of individual patients [129]. It is also possible to use other imaging biomarkers like OCT [130] or positron emission tomography [131].

DTs are crucial for enhancing joint decision-making, patient communication, and clinical decision-making in MS [132]. To prove their effectiveness and safety, they must be validated by research, professionals, and practical trials.

Data accuracy, privacy, and security are among the difficulties. The costly and intricate nature of DT development may make clinical practice monitoring more difficult. To ascertain which data contribute the most to prediction, assess that predictability, and incorporate this strategy into healthcare, more research is required.

Additionally, predictive models must be constructed. DTs can contribute to the realization of patient-centered care and precision medicine by examining every potential aspect of MS. In addition to improving patient well-being and diagnosis and monitoring, this will also save money, facilitate prevention, increase treatment alternatives, and empower people.

This may apply to MS and major chronic diseases worldwide. Further work is needed to build predictive models and understand which data contribute most to predictability.

### 7.2. Applications in Neuro-Surgery of Brain and Spine

DTs are virtual replicas of their physical counterparts and can help provide personalized surgical care. Chumnanvey et al. [133], via a systematic literature review of 25 studies, evaluated the efficacy and role of DTs in many phases of neurosurgical management. All 25 studies showed that digital twin applications have the potential to be more effective than conventional neurosurgical approaches when applied to brain and spinal surgery. Furthermore, the application of these new technologies could also lead to a reduction in post-operative complications.

It has been demonstrated that employing DT technology in neurosurgery can help alleviate some of the major problems that the field is currently facing, such as the shortage of trained surgical personnel, formal training requirements, and adequate instruments by which to address problems like intricate anatomical structures, inadequate ergonomics, and restricted maneuverability [134,135,136]. By creating a virtual model of a patient’s neuroanatomy and clinical profile using the sensor-rich, digitalized medical environment, DT increase the capacity to enhance treatment at all surgical management phases [137].

DTs can direct patient education and surgical planning prior to surgery, can enhance situational awareness and can prompt decision-making intraoperatively. Following surgery, these technologies allow for almost total digitalization for study, teaching, and record keeping [138,139]. Whereas cerebral lesions were not well characterized by previous imaging techniques, advancements in imaging technology enable surgeons to identify and view them with greater accuracy [140]. Target brain lesions can be seen and localized in real time during operational procedures thanks to the incorporation of neuronavigational devices in image-guided neurosurgery. These technologies are also used to determine the optimal interventional techniques and evaluate surgical risk [141]. The use of neuronavigational imaging reduces patient morbidity and death by enabling full spine or brain procedures to be performed without causing harm to nearby anatomical structures [142]. In addition, integrating robots with AI and ML insights into neurosurgical techniques shortens operating times while improving surgical results and minimizing complications [143].

The use of all these novel technologies has shown neurosurgeons how to develop a prediction model that allows them to target and generate trajectories, with the ability to produce damage control. Virtual reality (VR) and augmented reality (AR) technology have become increasingly used in neurosurgery recently. By utilizing AR and VR in pre-operative planning, neurosurgeons can enhance patient outcomes and better prepare for treatments. Because AR systems frequently superimpose preoperative scans or plans onto the patient’s anatomy without updating the model throughout the surgery, they have limits in terms of intraoperative guiding. Though VR systems are frequently utilized for surgical training simulations, their lack of real-time input means that they might not adequately depict real-world situations [144,145,146].

This review of the literature indicates that digital surgical treatments have produced better outcomes than conventional techniques. It is clear from the studies that were taken into consideration that a number of robotic techniques, including robotic navigational percutaneous pedicle screw placement, robotic trajectory guidance device, robotic guided platform for laser interstitial thermal ablation, and robotic stereo-electro-encephalography, are feasible for use in treating intracranial lesions such as tumors, spine surgery, and epilepsy surgery. The same is true of a number of navigated surgical techniques, such as computer-assisted surgical navigation for orbital wall fracture, navigation-based subcortical screw placement for lumbosacral spine surgery, navigation-based CT-guided navigation for percutaneous pedicle screw placement, navigation-based 3D fluoroscopy for treating degenerative conditions of the lumbar spine, and augmented reality surgical navigation for spine fixation surgery.

In order to view and duplicate patient phenotypes at various phases of the disease, healthcare teams may be able to rapidly query and utilize vast healthcare databases with the integration of DTs [147]. DTs are readily incorporated with other digital neurosurgery techniques, such as robotic, image-guided, and navigation-assisted procedures. Surgical situation awareness can be improved by DTs for thorough perioperative evaluation, surgical training, and dataset collection for the development of machine learning algorithms. These techniques have been used in several trials for a variety of brain and spine operations, which has improved surgical results and decreased complications [2,148,149,150,151,152,153].

Technological developments in AI and DTs have made it possible for surgeons to perform brain dissections with the assistance of robots [154]. These advancements have been applied in a variety of ways, such as robotic arms and assistants for stereotactic tasks, in studies to treat neurological conditions like epilepsy with better surgical results [155,156]. For image-guided neurosurgery, neuronavigation systems have also been incorporated [157]. Numerous neurological conditions, including foraminal stenosis, intra-axial brain lesions, arteriovenous malformations, and spinal stenosis, have been documented to be surgically treated by neuronavigation [158,159,160,161,162,163]. At least 23 of 25 studies have shown improved outcomes in the incorporation of novel digital neurosurgical procedures compared with traditional methods [164,165,166,167,168,169,170,171,172,173,174,175,176,177,178,179,180,181,182,183,184,185].

In conclusion, the observational research and randomized clinical trials included in this study all show better results when using DTs. DTs have enormous potential for managing all diseases, lowering surgical complications, and enhancing patient outcomes. Nevertheless, there is a need to keep building a higher caliber, longer-term, and more comprehensive body of scientific evidence.

## 8. Limitations

Despite the innovative potential of digital twins, their application in healthcare faces several limitations. One of the main limits lies in the collection and integration of heterogeneous data, such as genetic, clinical, behavioral, and environmental data. These data are often fragmented, incomplete, or come from non-interoperable sources, which compromises the creation of accurate models [186,187]. Furthermore, modeling human biological complexity, which requires the representation of dynamic processes such as metabolism, inflammation, or immune response, is extremely complex and may limit the accuracy of the digital twin [188]. Another significant obstacle is the high costs of the technological infrastructure needed to develop and manage digital twins, including computational resources and qualified experts. Added to this are the ethical and legal concerns related to the management of personal data, which require robust solutions to ensure privacy, security, and regulatory compliance [189]. Finally, acceptance by healthcare professionals and patients remains a challenge, as many are still skeptical about the reliability and applicability of these technologies in daily clinical practice.

## 9. Future Directions

To overcome current limitations, future directions of digital twin technology in healthcare focus on several key areas. The integration of artificial intelligence and machine learning will enable the analysis of large amounts of complex data more efficiently, improving the predictive capacity and personalization of models [190]. The development of wearable devices and advanced sensors will enable continuous collection of data in real time, providing more accurate and up-to-date information on the patient’s health status. In parallel, the standardization of formats and protocols for data management and exchange will foster interoperability between different platforms and systems, making digital twins more accessible and useful. Another area of growth is the simulation of personalized therapies and interventions, which could reduce clinical trial times and improve therapeutic outcomes [191]. Furthermore, more advanced policies and regulations will be needed to ensure data security and address ethical issues. Finally, collaboration between academic institutions, technology companies and healthcare systems will accelerate large-scale adoption, promoting the training of healthcare professionals to best exploit this technology in clinical practice [192].

## 10. Conclusions

We thought it would be helpful to describe, in a narrative review, the technological innovations and clinical applications of greatest interest in medicine that have emerged over the past 20 years in a rapidly expanding sector. We attempted to conduct a fairly critical review of the most recent and pertinent global literature, analyzing any points of agreement or contrast, and we also tried to provide what information might be sufficient to change clinical practice with this technology. In this regard, we attempted to address some questions in order to contribute a more thorough evaluation of the subject to the literature, including: 1. What requirements must a digital twin meet, particularly with regard to trustworthiness and usability? 2. What level of accuracy is necessary for a digital twin to be effective? 3. What benefits must a digital twin offer in order to warrant its creation? Regretfully, we still lack conclusive solutions in medicine, in contrast to industrial models. There are significant regulatory constraints and barriers (as described in the previous paragraphs), and successful commercialization tactics remain mostly unexplored. The biological heterogeneity of patients is one of the major obstacles to precision medicine and the use of MDTs, and, given our understanding of human biology and the data required to capture it, we will probably need to develop higher resolution models than are currently available to account for this. As previously mentioned, in many cases, MDTs will need to develop additional standards and requirements for approval. Once we overcome these obstacles, medical digital twins will revolutionize healthcare, assisting us in the transition from curative to preventive medicine.

## Figures and Tables

**Table 1:** The knowledge and application of DTs in the various fields referenced in the manuscript.

Field	What is Known	Applications of Digital Twin Technologies
Healthcare (general)	Digital twins are virtual replicas of physical entities, enabling real-time simulation and prediction. They integrate data from IoT devices, EMRs, and sensors to provide insight	- Personalized treatment planning - Predictive modeling for disease progression - Optimizing clinical workflows
Oncology	Cancer treatment often requires personalized approaches due to high variability in tumor behavior and patient responses	- Simulating tumor growth and response to treatments - Predicting efficacy of chemotherapy or immunotherapy - Optimizing radiation doses
Cardiology	Cardiovascular diseases are complex, involving multiple interacting factors like blood flow, heart rhythm, and vessel health	- Modeling patient-specific heart dynamics - Predicting the outcome of surgeries like valve replacements - Monitoring and optimizing pacemakers
Neuroscience	Chronic Diseases: Neurological disorders like Multiple Sclerosis, Parkinson’s, Alzheimer’s, and epilepsy have complex mechanisms and high individual variability Neurosurgery: Neurosurgical procedures require precision in understanding brain structures and functional regions	Chronic Diseases: - Modeling disease progression (e.g., Alzheimer’s protein accumulation) - Simulating responses to treatments like neurostimulation or medication - Personalized management plans for conditions like epilepsy Neurosurgery: - Pre-surgical planning by simulating brain anatomy and activity - Real-time adjustments during surgeries to avoid damage to functional areas - Predicting recovery outcomes based on patient-specific data

## Data Availability

No study subjects or cohorts have been reported in previous studies.
